# Riding out the storm: depleted fat stores and elevated hematocrit in a small bodied endotherm exposed to severe weather

**DOI:** 10.1093/conphys/coad011

**Published:** 2023-03-20

**Authors:** N E Freeman, M Gustafson, T J Hefley, W A Boyle

**Affiliations:** Division of Biology, Kansas State University, 116 Ackert Hall, Manhattan, KS 66506, USA; School of Natural Sciences, Bangor University, Deiniol Road, Bangor, Gwynedd, LL57 2DG, UK; Division of Biology, Kansas State University, 116 Ackert Hall, Manhattan, KS 66506, USA; Department of Biological Sciences, Boise State University, 2133 Cesar Chavez Lane, Boise, ID 83725, USA; Department of Statistics, Kansas State University, 101 Dickens Hall, Manhattan, KS 66506, USA; Division of Biology, Kansas State University, 116 Ackert Hall, Manhattan, KS 66506, USA

**Keywords:** QMR, precipitation, lean mass, fat stores, energetics, body composition

## Abstract

In the mid-continental grasslands of North America, climate change is increasing the intensity and frequency of extreme weather events. Increasingly severe storms and prolonged periods of elevated temperatures can impose challenges that adversely affect an individual's condition and, ultimately, survival. However, despite mounting evidence that extreme weather events, such as heavy rain storms, can impose short-term physiological challenges, we know little regarding the putative costs of such weather events. To determine the consequences of extreme weather for small endotherms, we tested predictions of the relationships between both severe precipitation events and wet bulb temperatures (an index that combines temperature and humidity) prior to capture with body composition and hematocrit of grasshopper sparrows (*Ammodramus savannarum*) caught during the breeding season at the Konza Prairie Biological Station, Kansas, USA, between 2014 and 2016. We measured each individual's fat mass, lean mass and total body water using quantitative magnetic resonance in addition to their hematocrit. Individuals exposed to storms in the 24 hours prior to capture had less fat reserves, more lean mass, more water and higher hematocrit than those exposed to moderate weather conditions. Furthermore, individuals stored more fat if they experienced high wet bulb temperatures in the week prior to capture. Overall, the analysis of these data indicate that extreme weather events take a physiological toll on small endotherms, and individuals may be forced to deplete fat stores and increase erythropoiesis to meet the physiological demands associated with surviving a storm. Elucidating the potential strategies used to cope with severe weather may enable us to understand the energetic consequences of increasingly severe weather in a changing world.

## Teaser

Understanding how organisms survive extreme weather events is important as they are predicted to increase in severity. Grasshopper sparrows exposed to storms had depleted fat stores, retained more water, and had elevated hematocrit supporting the idea that surviving storms is energetically costly.

## Introduction

Global variation in climate has had a profound influence on broad-scale patterns of species distribution via selection on traits that mediate organismal responses to weather (Pearson and Dawson, 2003; Kearney and Porter, 2009). While climates have changed over the evolutionary histories of all extant species, the current rates of change are unprecedented (Alley *et al.,* 2003; Smith *et al.,* 2015). Consequently, species living in the modern world are coping with conditions that may be at the limits of those under which they historically thrived (O’Neill and Oppenheimer, 2004). The majority of studies investigating species-level responses to changing climates have focused on rising temperatures and have documented associated poleward range shifts (MacLean and Beissinger, 2017), shifts in phenology (Renner and Zohner, 2018; Piao *et al.,* 2019) or changes in performance (Deutsch *et al.,* 2008; Román-Palacios and Wiens, 2020). Importantly, climate change is far more complex than just increasing temperatures. As the world warms, precipitation regimes are also affected with some regions becoming drier and others becoming wetter (IPCC, 2022). Furthermore, altered patterns of temperature, humidity and rainfall are leading to widespread changes in the timing, severity and intervals between rain storms (IPCC, 2022). For example, mid-continental regions such as the Great Plains of North America are characterized by substantial inter- and intra-annual variation in both temperature and precipitation (Cherwin and Knapp, 2012; Ojima *et al.,* 2021). Projections for this region are for a mean temperature increase of 4.4°C to 6.6°C by 2099 and the number of days per year where temperatures exceed 32°C is predicted to quadruple (Vose *et al.,* 2017). Total precipitation is predicted to decline by 5% to 10% (Easterling *et al.,* 2017), leading to more prolonged periods of drought but also higher-intensity precipitation events (Janssen *et al.,* 2014; Hicke *et al.,* 2022).

For endothermic animals such as birds, the effects of temperature on individual physiology, behaviour and survival are relatively well studied. Endotherms maintain stable core body temperatures and when ambient temperatures exceed the upper and lower limits (i.e. upper and lower critical temperatures) of their thermoneutral zone, their metabolism increases (Scholander *et al.,* 1950). A large suite of traits define the range of temperatures under which each species can maintain low energy expenditure including body size, conductance and metabolic rates (Mitchell *et al.,* 2018; Gerson *et al.,* 2019; McKechnie and Wolf, 2019). When faced with elevated ambient temperatures that exceed the upper limit of their thermoneutral zone, endotherms can cope behaviourally by seeking cooler microclimates (Etches *et al.,* 2008), ceasing or reducing activities that generate excess body heat (Silva *et al.,* 2015) and actively cooling via evaporative water loss (Lasiewski *et al.,* 1966), leading to increased water intake to battle dehydration (May and Lott, 1992).

Our understanding of how endotherms respond to precipitation is substantially less complete, but there is a growing number of examples that link behaviour, physiology and demography to either too little or too much rain. Under drought conditions, birds frequently experience lower reproductive success (Skagen and Adams, 2012; Colón *et al.,* 2017), adjust movement (Bateman *et al.,* 2015) or exhibit altered abundance and patterns of occupancy (Albright *et al.,* 2017; Cady *et al.,* 2019). Responses to rainfall may reflect direct challenges to maintain internal homeostasis or may be the result of indirect bottom-up (e.g. vegetation and habitat structure, prey communities) or top-down (e.g. predator communities) processes (Boyle *et al.,* 2020). Extreme precipitation events can also influence fitness directly. Storms are weather events lasting from hours to days with heavy rain, frequently accompanied by high winds, lightning and thunder. During and immediately following storms, some birds suffer nest failure (Conrey *et al.,* 2016), alter timing or investment in breeding activities (Mahony *et al.,* 2006), move away from affected regions (Ramos, 1989; Boyle *et al.,* 2010), alter their physiology and body condition (Wingfield *et al.,* 1998, 2017; Krause *et al.,* 2018) and sometimes, die (Newton, 2007; Wellicome *et al.,* 2014).

While direct costs of rain on birds can be independent of temperature, other potential costs result from interactions between temperature and humidity. At high temperatures, birds expend energy to maintain stable internal temperatures via evaporative cooling (Lasiewski *et al.,* 1966; Pollock *et al.,* 2021). But, the effectiveness (i.e. energy cost) of evaporative cooling is dependent upon the strength of the moisture gradient between the air and mucous membranes such as the mouth or gular regions, where drier conditions assist with evaporation (Lasiewski *et al.,* 1966; van Dyk *et al.,* 2019). Therefore, under humid conditions, birds suffer greater thermoregulatory costs and are at greater risk of fatal consequences of high temperatures (Gerson *et al.,* 2014; McKechnie and Wolf, 2019). Thus, measures of environmental conditions that take humidity into account, such as wet bulb temperature, are far more biologically relevant to birds and have greater predictive power to explain broad associations between climate and distributions than the commonly used measure of temperature (i.e. dry bulb temperature; James, 1970).

Birds cope with severe weather variability by triggering an ‘emergency life history stage’ (Wingfield *et al.,* 1998; Wingfield, 2013). When faced with challenges, vertebrates, including birds, activate hormonal pathways that result in the release of a suite of hormones including catecholamines (e.g. epinephrine) and glucocorticoids into the bloodstream, which mobilize energy stores and influence body composition (reviewed in Sapolsky *et al.,* 2000; Wingfield *et al.,* 2017). Fat is the component of body composition that provides the greatest amount of energy per unit mass (Jenni and Jenni-Eiermann, 1998), and in birds, can be rapidly deposited and mobilized in response to short-term foraging excesses or deficits (Dick *et al.,* 2020). While fat deposition or mobilization can be regulated within minutes, body composition responses are typically evident within hours to days (Seewagen and Guglielmo, 2010; Boyle *et al.,* 2012; Krause *et al.,* 2017). Birds can also facultatively modulate components of lean mass (i.e. muscles and organs; (Guglielmo and Williams, 2003; Gerson and Guglielmo, 2011a), but gram for gram, mobilization of lean mass yields less energy than fat. Carbohydrates, fat and protein can be used to increase fat stores, while lean mass growth requires a protein rich diet (Guglielmo *et al.,* 2022), and thus, lean mass may respond to environmental stressors more slowly than fat mass due to the need of a more protein-rich diet. Depleted lean mass typically represents either a weight-saving strategy or a source of metabolic water during flight, or more severe energy depletion under starvation conditions (Karasov and Pinshow, 1998; Gerson and Guglielmo, 2011b).

Changes in weather may also impact an individual's ability to maintain water homeostasis and, as a result, hematocrit. Water does not provide metabolic energy but is required for functions from molecular to organ level scales (Gerson and Guglielmo, 2011a). Animals may experience extreme dehydration leading to the shutdown of metabolic pathways and organs, and ultimately, death (e.g. Albright *et al.,* 2017). Elevated temperatures may lead to dehydration resulting in more concentrated blood (i.e. higher hematocrit; Fair *et al.,* 2007). To combat dehydration, birds may drink or consume foods rich in water (Bartholomew and Cade, 1963). Thus, rainfall may allow individuals to maintain water balance. Alternatively, individuals may endogenously produce water through the catabolism of fat (Rutkowska *et al.,* 2016) and protein (Jenni and Jenni-Eiermann, 1998; Gerson and Guglielmo, 2011a). Maintenance of water balance allows for viscosity of blood to be maintained ensuring proper circulation and the oxygen transport. Understanding the interplay between water balance and blood is important because elevated hematocrit is associated with migratory behaviour (Krause *et al.,* 2016a) and energy expenditure (Yap *et al.,* 2019) and has downstream consequences on survival (Bowers *et al.,* 2014) including during winter and extreme weather (Fair *et al.,* 2007; Krause *et al.,* 2016b).

We quantified body composition and hematocrit of adult grasshopper sparrows (*Ammodramus savannarum*) during three breeding seasons at the Konza Prairie Biological Station in Kansas, USA. The grasshopper sparrow is a small, ground-nesting passerine whose survival and abundance appear to be strongly influenced by weather on both breeding and non-breeding areas (Gorzo *et al.,* 2016, Macías-Duarte *et al.,* 2017, Silber *et al.,* 2023). Thus, in order to survive rainstorms and periods of hot, humid weather, we expected birds to therefore modulate body composition and hematocrit to meet the energetic demands of inclement weather. Storms introduce foraging uncertainty that can lead to short-term fasting, so we predicted that individuals would deplete fat and protein and therefore have lower fat and lean mass following a storm. Alternatively, rain may allow for rehydration, in which case total body water and therefore, lean mass would increase. Following storms, we also expected hematocrit to increase to meet increased energy expenditure (Fair *et al.,* 2007). Depending if temperatures are within a tolerable range, fat or lean mass may remain constant or even increase. However, if temperatures exceed the thermoneutral zone, we predicted that individuals would then have lower fat and lean mass because energy would be required to maintain the higher metabolic demand associated with thermoregulation. Declines in fat and lean mass could also allow individuals to cope with heat stress by lowering their metabolic heat load (Klaassen *et al.,* 1990). Body water and hematocrit responses also provide insight into whether individuals are capable of maintaining key elements of homeostasis or not under current weather conditions. Under hot conditions, which can lead to dehydration via evaporative water loss, we expected body water to decrease and hematocrit to increase.

## Methods

### Study site and species

We conducted our study at the Konza Prairie Biological Station (hereafter ‘Konza Prairie’, 39°05′ N, 96°35′ W) in northeastern Kansas, USA. Konza Prairie is a native tallgrass prairie composed of experimentally manipulated watersheds with replicated combinations of grazing (ungrazed or grazed by bison or cattle) and burning frequency (1-, 2-, 3-, 4-, or 20-year intervals) treatments. The weather at Konza Prairie is highly dynamic with a mean annual temperature of 12°C and a mean annual precipitation of 835 mm (Goodin *et al.,* 2003). Precipitation varies considerably inter- and intra-annually with the majority of rainfall occurring in May, June and September (Goodin *et al.,* 2003).

We captured grasshopper sparrows at Konza Prairie throughout their breeding season (April–August). Grasshopper sparrows are small songbirds that breed in the grasslands of North America, occupying in patchy grasslands with few shrubs (Powell, 2008; Vickery, 2020). Throughout the breeding season, individuals devote energy to finding mates, building nests, incubating eggs (female only) and feeding young in addition to avoiding predators and surviving inclement weather (Vickery, 2020). Breeding pairs may attempt raising one to three broods per season due to high rates of nest failure (Vickery, 2020). Furthermore, individuals may disperse within the breeding season. For example, 75% of male grasshopper sparrows changed territories, moving up to 8.9 km (Williams and Boyle, 2018).

All work was conducted under approved ethical animal care and use protocols (Kansas State University #3260) and research permits from the North American Bird Banding Laboratory (#23836), Konza Prairie Biological Station and the Kansas Department of Wildlife, Parks and Tourism.

### Field methods

Throughout the breeding seasons of 2014–16, we caught adult grasshopper sparrows using mist nets. We marked birds using a unique combination of three coloured leg bands and one US Fish and Wildlife Services issued aluminium, numbered leg band. Individuals were sexed based on the presence of a cloacal protuberance (male) or a brood patch (female) and were weighed. Using a mobile quantitative magnetic resonance (QMR) machine (Echo-Medical Systems, Houston, TX, USA), we estimated body composition of the individual (n = 325) by measuring fat mass (g), total body water (g) and lean mass (g) prior to release. Fat mass reflects the amount of fat the bird has stored in addition to lipids in cellular membranes. Total body water is an estimate of the mass of all water in the tissues, including blood and any water in the digestive and urinary systems. Lean mass estimates the mass of organs and tissues but also includes water (also known as wet lean mass, Boyle *et al.,* 2012). QMR has previously been used to understand relationships between body composition, energetics and migration in birds and bats (e.g. McGuire *et al.,* 2018; Kelsey *et al.,* 2019; Guglielmo *et al.,* 2022). Furthermore, the use of QMR to quickly and accurately quantify the body composition of live animals has been validated and is repeatable with < 3% coefficients of variation for each measure (Guglielmo *et al.,* 2011).

### Hematocrit

We collected a ~ 70 μl blood sample from 263 of the 325 individuals that were scanned in the QMR machine. Blood was drawn from the brachial vein using a 26-gauge needle, collected in a capillary tube, and kept it on ice for up to ~ 6 hours. We centrifuged capillary tubes at 14 000 rpm for 5 minutes, which separated the red blood cells from the plasma, platelets and white blood cells. Hematocrit was measured as the percentage of the blood made up of packed red blood cells.

### Weather metrics

Air temperature (°C), relative humidity (%) and precipitation (mm) were measured using a Campbell Scientific (CR-10) data logger located at the Konza Prairie Biological Station (Nippert, 2022). Air temperature and relative humidity were recorded on the hour and precipitation was recorded every 15 minutes. Using equation (1), we calculated the wet bulb temperature (°C) for each hour (Stull, 2011).


(1) \begin{align*} {T}_w&=T\ \mathrm{atan}\left[0.151977{\left(\mathrm{RH}\%+8.313659\right)}^{1/2}\right]\notag\\&\quad+\mathrm{atan}\left(T+\mathrm{RH}\%\right)- \mathrm{atan}\left(\mathrm{RH}\% - 1.676331\right)\notag\\ &\quad+0.00391838{\left(\mathrm{RH}\%\right)}^{3/2}\mathrm{atan}\left(0.023101\mathrm{RH}\%\right)\notag\\&\quad- \mathrm{4.686035.} \end{align*}

Wet bulb temperature (*T_w_*) is a function of temperature (*T*) and relative humidity (RH%) and is used as a measure of heat stress (e.g. Kang *et al.,* 2020) because it takes into account how heat dissipation and evaporative cooling can differ based on both temperature and humidity. Furthermore, wet bulb temperature explains size variation in birds better than air temperature (James, 1970). To assess the relationship between body composition and weather in the week leading up to capture, we calculated the average wet bulb temperature in the 168 hours (= 1 week) prior to the capture time of each individual. We measured the average wet bulb temperature across a weeklong period because we were interested in the cumulative effects of many hot, humid days in a row.

To assess the relationship between storms, body composition and hematocrit, we first characterized precipitation events. The start of a precipitation event was the first of the 15-minute periods where rainfall was detected and continued through all periods recording measurable precipitation. Events ended at the time when no rain was detected in eight consecutive 15-minute periods (i.e. 2 hours) following the last measured precipitation. We then classified precipitation events as storms if the total precipitation during the event was greater than one standard deviation above the mean amount of precipitation that fell during all rainfall events in the study (similar to the > 10 and > 20 mm thresholds for heavy and strong rainstorms in Skagen and Adams, 2012, Öberg *et al.,* 2015). Based on the capture time of the individual, we then determined whether an individual experienced a storm in the 48 hours prior to capture.

### Statistical methods

To identify whether weather prior to capture affected body composition and hematocrit, we used four generalized additive models (GAM) with fat mass, total body water, lean mass and hematocrit as response variables. Fat mass, total body water and lean mass were modelled with a Gaussian distribution while hematocrit was modelled with a beta distribution because it is a percentage. We used total body mass (to account for the variation in body composition by size), sex (male or female), whether a storm occurred in the 48 hours prior to capture (0 = no storm, 1 = storm), the average wet bulb temperature in the week leading up to capture, and the ordinal date (to account for seasonal changes in weather and body composition) as predictor variables in all four models. The continuous variables (total body mass, average wet bulb temperature and ordinal date) were included as smooth terms to account for non-linear relationships with each response variable. For example, the average wet bulb temperature was included as a smooth term because hot and cold extremes could be detrimental to energy stores while ordinal date was included as a smooth term because body composition and hematocrit may vary non-linearly across the breeding season.

To explore the sensitivity of our results to alternative modelling approaches, we repeated our modelling efforts while varying how we controlled for body size. We compared models that included total body mass, tarsus and both total body mass and tarsus to models with no control for body size using AICc (methods outlined in the Supplemental materials). We report the results of the models that included total body mass below because the top models consistently contained mass as a predictor. Models that included tarsus performed similarly to models that contained no measure of body size or mass (Supplementary Tables S1 and S2).

We used the package ‘mgcv’ (Wood, 2011, 2017) to perform the analysis in R (version 4.1.1, R Core Team 2021).

## Results

We captured grasshopper sparrows from early May to the end of July in 2014–16 (earliest capture date, May 2; latest capture date, July 29). A total of 325 individuals were captured of which, 48 were female and 277 were males. Individuals had an average total body mass (± standard deviation [SD]) of 17.15 ± 0.86 g (range = 14.94–20.16 g) and total body mass was constant throughout the breeding season (Pearson’s correlation: r = −0.08, *P* = 0.17). Grasshopper sparrows had an average fat mass of 0.64 ± 0.23 g (range = 0.04–1.58 g), an average total body water of 11.98 ± 1.58 g (range = 0.28–17.72 g) and an average lean mass of 14.08 ± 0.84 g (range = 8.09–16.29 g). Hematocrit ranged between 40% and 59% with a mean of 50.05 ± 3.02%.

During the breeding season, the wet bulb temperature ranged from −4.88°C to 28.92°C (mean ± SD in 2014 = 15.20 ± 6.89°C, 2015 = 16.47 ± 6.23°C and 2016 = 16.35 ± 6.43°C) and increased throughout the breeding season (Pearson’s correlation: r = 0.67, *P* < 0.001). Total rainfall during the breeding season varied substantially between the 3 years (2014 = 343.80 mm, 2015 = 515.80 mm, 2016 = 464.20 mm) and was unpredictable within the breeding season (Pearson's correlation: r = −0.07, *P* = 0.24). Rain falling in individual precipitation events ranged from 0.30 to 96.60 mm (mean = 6.68 ± 11.53 mm) with events lasting between 15 minutes (the shortest detectable time) and 24.5 hours. Precipitation events were considered storms if rainfall exceeded 18.21 mm. Across the 3 years, there were 62 individuals that experienced a storm in the 48 hours prior to capture. In total, there were 18 storms that occurred during the three breeding seasons (May–July; 2014 = 5, 2015 = 8, 2016 = 5 storms), 10 of which occurred within 48 hours prior to a capture event.

### Predictors of body composition and hematocrit

**Figure 1:**
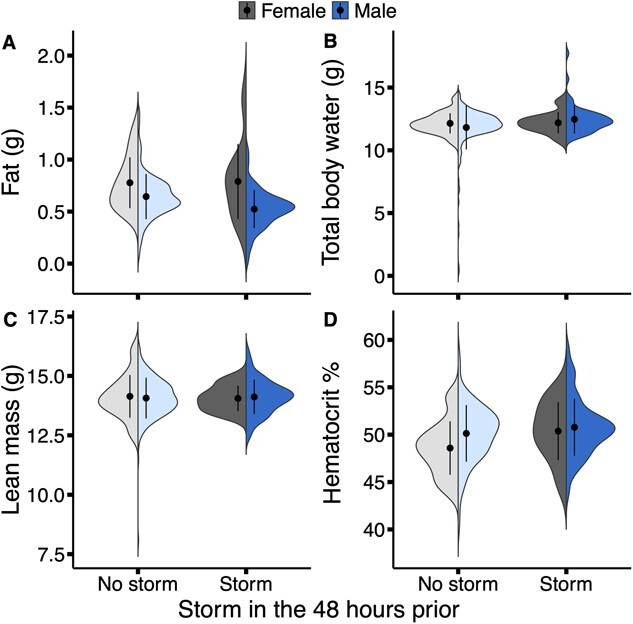
The relationship between whether there was a storm in the 48 hours prior to capture (yes, darker shade; no, lighter shade) and (A) fat mass, (B) total body water, (C) lean mass and (D) hematocrit of male (blue) and female (grey) grasshopper sparrows (*A. savannarum*, *n* = 325 for A, B and C, *n* = 263 for D). The black points represent the mean and the whisker represents the standard deviation for each sex.

Grasshopper sparrows that were exposed to storms in the 48 hours prior to capture on average had 0.08 g less fat (± 0.03 SE, *P* = 0.01), 0.44 g more water (± 0.17 SE, *P* = 0.01) and 0.15 g more lean mass (± 0.07 SE, *P* = 0.03) than those that did not recently experience a storm (Table 1, Fig. 1A–C). Similarly, a storm prior to capture was also associated with higher hematocrit (0.03 ± 0.02 SE, *P* = 0.05, Table 2, Fig. 1D).

**Table 1:** Output from three generalized additive models (family = Gaussian, link = identity) used to assess the relationship between the body composition of 325 grasshopper sparrows (*A. savannarum*) and whether an individual experienced a storm prior to capture

	Fat mass			Total body water			Lean mass		
Parametric parameters	Estimate ± SE	t value	*P*	Estimate ± SE	t value	p value	Estimate ± SE	*t* value	*P*
Intercept	0.77 ± 0.03	24.66	< 0.001	11.80 ± 0.18	64.31	< 0.001	14.01 ± 0.07	189.79	< 0.001
Sex	−0.12 ± 0.03	−3.73	< 0.001	0.11 ± 0.20	0.58	0.56	0.06 ± 0.08	0.72	0.47
Storm pre-capture	−0.08 ± 0.03	−2.80	0.01	0.44 ± 0.17	2.58	0.01	0.15 ± 0.07	2.21	0.03
**Smooth parameters**	**edf**	**F**	**P value**	**edf**	**F**	**p value**	**edf**	**F**	**p value**
Mass	3.06	13.30	< 0.001	1.00	79.68	< 0.001	3.67	139.35	< 0.001
Temperature	1.00	5.55	0.02	1.00	1.66	0.20	1.00	0.07	0.79
Ordinal date	1.00	0.02	0.89	7.92	17.28	< 0.001	2.22	2.74	0.05

The three response variables (fat mass (g), total body water (g) and lean mass (g)) were measured using a quantitative magnetic resonance machine. Parametric predictors in each model included the sex of the individual and whether the individual experienced a storm prior to capture (binary: no or yes). Smooth term predictors included the total body mass (g) of the individual, the average wet bulb temperature in the week leading up to capture, and the ordinal date of capture. SE = standard error and edf = effective degrees of freedom.

**Table 2:** Output from a generalized additive model (family = beta, link = logit) used to assess the relationship between hematocrit of 263 grasshopper sparrows (*A. savannarum*) and whether an individual experienced a storm prior to capture

Parametric parameters	Estimate ± SE	*z* value	*P*
Intercept	−0.03 ± 0.02	−1.54	0.12
Sex	0.03 ± 0.02	1.40	0.16
Storm pre-capture	0.03 ± 0.02	1.93	0.05
**Smooth parameters**	**edf**	**Chi** ^**2**^	**P value**
Mass	1.00	0.77	0.38
Temperature	1.82	1.69	0.56
Ordinal date	3.11	10.72	0.02

The response variable was hematocrit (bounded by 0 and 1). Parametric predictors in each model included the sex of the individual and whether the individual experienced a storm prior to capture (binary: no or yes). Smooth term predictors included the total body mass (g) of the individual, the average wet bulb temperature in the week leading up to capture, and the ordinal date of capture. SE = standard error and edf = effective degrees of freedom.

**Figure 2:**
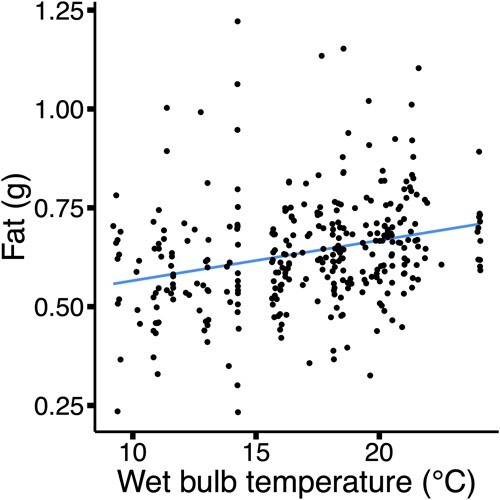
The relationship between the average wet bulb temperature (°C, wet bulb temperature is an index of temperature and humidity) in the week prior to capture and the fat mass (g) of grasshopper sparrows (*A. savannarum*). Each point represents one individual (*n* = 325).

Individuals exposed to elevated wet bulb temperatures in the week prior to capture had larger fat stores (*P* = 0.02) than individuals exposed to more moderate temperatures (Fig. 2). As the breeding seasons progressed, individuals held more water (*P* < 0.001), had a higher lean mass (*P* = 0.05; Table 1) and had lower hematocrit (*P* = 0.02; Table 2). Females had 0.12 g more fat than males (± 0.03 SE, *P* < 0.001), but the sexes did not differ in body water, lean mass or hematocrit (Tables 1 and 2).

## Discussion

Using 3 years of physiological data collected on wild birds, we provide evidence that individuals modulate their body composition and hematocrit following exposure to severe weather. Individuals that survived and were captured after storms carried less fat, retained more water and had higher hematocrit than individuals who experienced less extreme weather prior to capture. In avian studies, fat is primarily viewed as the energy source used to fuel long, migratory flights (Deppe *et al.,* 2015; Araújo *et al.,* 2019). Our work suggests that fat deposits may also be used to cope with short-term, more acute stressors such as extreme precipitation events. This implies that surviving a storm is energetically costly and weather possibly limits foraging options to the extent that individuals resort to catabolism of their fat stores to meet thermoregulatory and other metabolic demands.

The high energy expenditure associated with surviving a storm may also explain why hematocrit was elevated in storm-exposed individuals. High hematocrit has previously been associated with increased energy demands during migration (Krause *et al.,* 2016a), winter acclimatization (reviewed by Fair *et al.,* 2007) and reproduction (Lownie *et al.,* 2022). We suggest hematocrit may even be modulated to assist with surviving severe weather via two potential mechanisms. First, to survive storms, birds must thermoregulate during wet and damp conditions, potentially even with wet feathers. To meet the energetic and oxygen demands of thermogenesis, birds can quickly upregulate erythropoiesis and modulate their hematocrit by producing and releasing reticulocytes (young red blood cells) into the bloodstream (Campbell and Ellis, 2013). Reticulocytes are larger than erythrocytes (Campbell and Ellis, 2013) and can therefore lead to elevated hematocrit. Alternatively, elevated hematocrit could also indicate that the birds were dehydrated post-storm. Dehydration leads to a larger proportion of the blood being composed of red blood cells and more viscous blood due to reduced plasma volume (Zhou *et al.,* 1999). However, total body water was higher in birds exposed to a storm so dehydration is less likely as an explanation for elevated hematocrit following a storm. Overall, while high hematocrit can mean an increased aerobic capacity, if hematocrit is too high, it could make birds and other endotherms susceptible to extreme heat due to a decreased ability to manage internal temperatures via heat loss (Zhou *et al.,* 1999).

How endotherms, including birds, respond to elevated temperatures is relatively well understood. Here, we build on previous results of the relationship between temperature and avian physiology by considering how temperature and humidity work together (i.e. wet bulb temperature). Grasshopper sparrows that experienced warmer wet bulb temperatures in the week prior to capture had more fat than those that experienced cooler weather. Warmer temperatures may allow for more foraging opportunities with increased prey activity and availability (e.g. Asmus *et al.,* 2018), enabling individuals to grow and maintain fat stores. Throughout the majority of our study, the temperatures that birds experienced were well within their thermoneutral zone (estimated upper limit, 38.7°C and heat tolerance limit, 45.2°C for temperate birds; Pollock *et al.,* 2021). The maximum average wet bulb temperature during this study was 28°C and the maximum air temperature recorded was 39.48°C. The days where air temperatures potentially exceeded the thermoneutral zone were few: 6 days in 2014, 0 days in 2015, and 3 days in 2016. Thus, it is unlikely that we would observe individuals who were depleting their energy stores as a result of thermoregulatory stress. Furthermore, wet bulb temperature was not associated with differences in total body water or hematrocrit, further suggesting that elevated temperatures did not lead to dehydration.

In our analyses, we controlled for ordinal date to account for intra-annual changes in temperature and precipitation. Throughout the breeding season, total body water and lean mass increased, hematocrit declined and total body mass and fat mass did not vary over time. The observed shift in total body water and lean mass but not fat mass may be a result of increasing temperatures, declining water availability, or shifts reproductive behaviour and therefore energy expenditure throughout the season. Individuals may respond to drier conditions by storing more water endogenously (e.g. Bartholomew and Cade, 1963), leading to higher body water and potentially wet lean masses. Alternatively, lean mass may increase throughout the summer because energy stores could have been depleted following spring migration but rebuilt in time for fall migration. However, because birds are capable of rebuilding fat and lean mass quickly (days to weeks instead of over an entire season) particularly prior to migration, and because we did not see a correlation between ordinal date and fat mass nor total body mass, this explanation is less likely. Additionally, our results contradict previous work on the energetic stress hypothesis that mass, and therefore, fat and lean mass should decrease throughout breeding (Ricklefs, 1974; Merila and Wiggins, 1997; Boyle *et al.,* 2012). However, because we do not know where each individual was in the timing of their breeding activities, and because the grasshopper sparrows may re-nest several times within a single breeding season, we cannot draw connections between the energetics of reproduction and our measures of body composition.

Contrary to our prediction, birds exposed to storms had higher lean mass than birds that experienced average weather conditions. Lean mass, composed of muscles and organs, represents another source of energy that birds may rely upon to survive. Thus, we predicted that individuals may break down proteins for energy and have lower lean mass following severe weather. We suspect that because our estimate of lean mass also includes a large proportion of water content (Boyle *et al.,* 2012), our observation of elevated total body water following a storm is most likely driving higher lean mass. Alternatively, survivor bias could be skewing results whereby individuals with lower lean mass either did not survive the severe weather, or they dispersed to avoid exposure. Another non-mutually exclusive explanation could be that individuals either prepare for, or following a storm, try to recoup losses in fat stores by foraging more and thus have fuller gastrointestinal tracts. However, passage of food through the gastrointestinal tracts of birds is rapid (1–2 hours in several species of sparrow; Stevenson, 1933), that is why the timing of the estimation of body composition post-storm is important. A study at a finer scale than our 48-hour window is necessary to elucidate the relationship between storms and lean mass and an immediate measure of body composition post-storm would provide a better indication of how individual's energy stores are used to survive extreme weather.

Our results indicate that individuals may modulate their body composition and hematocrit following both an acute, severe precipitation event and a more prolonged span of elevated temperature and humidity. This suggests that individual body composition, in particular fat stores, are sensitive to a range of environmental conditions. Historically, most work investigating avian fat stores, lean mass and hematocrit are conducted in the winter (Houston and McNamara, 1993; Bednekoff and Houston, 1994) or during migration (King and Farner, 1965; Cooper *et al.,* 2015). Focusing on more rapid changes in body composition (e.g. hours or days) would complement past 0research examining longer term changes in body composition (e.g. across entire seasons) and could be achieved by repeatedly sampling individuals pre- and post-storms. Expanding these studies to other periods of the annual cycle and different temporal scales could help to elucidate how individuals augment their body composition in response to a wider range of environmental conditions and acute stressors to better predict individual performance.

The rise in global temperatures and shifts in the frequency and intensity of precipitation (IPCC 2022) has consequences and may push wildlife towards their physiological limits to the detriment of fitness. Such consequences are already present where breeding birds in arid regions are no longer able to maintain their own condition let alone provision offspring (e.g. Silva *et al.,* 2015; van de Ven *et al.,* 2019). Here, we show that high temperatures, reduced total precipitation and increased severity of storms may impact small-bodied endotherms body composition and their aerobic capacity. Interestingly, our results suggest that water availability can play an important role in how individuals respond to their abiotic environment. Taken together with previous research on grassland birds, it highlights the need to take an integrative approach and consider a range of physiological and environmental conditions that can influence not only behaviour, but also individual fitness.

## Authors contributions

N.E.F., M.G., T.J.H. and W.A.B. conceived the ideas; M.G. and W.A.B. collected the data; N.E.F. analysed the data; N.E.F. led the writing of the manuscript. All authors contributed critically to revising the manuscript and gave final approval for publication.

## Funding

This work was supported by the National Science Foundation [DEB-1754491 to W.A.B. and T.J.H.] and a Postdoctoral Research Fellowship from the American Association of University Women [to N.E.F.].

## Data availability

The weather data underlying this article is available online (Nippert, 2022 DOI: 10.6073/pasta/432124c318000539cc44a76ba27eef94). The avian body composition and hematocrit data are accessible upon request from the corresponding author.

## Supplementary Material

Web_Material_coad011
