# Supplementary material for: Riding out the storm: depleted fat stores and elevated hematocrit in a small bodied endotherm exposed to severe weather
Source: Conserv Physiol. 2023 Mar 20;11(1):coad011. doi: 10.1093/conphys/coad011 (PMC10026549; doi:10.1093/conphys/coad011)
Supplement: Web_Material_coad011 [file web_material_coad011.pdf]

Supporting materials for Freeman et al. (2023) ***Riding out the storm: depleted fat stores and elevated hematocrit in a small bodied endotherm exposed to severe weather***

Inclusion of total body mass as a predictor of body composition

Methods

In analyses where the response variable is a measure of an individual's mass or component of mass, it is common to include morphometric measurements to account for variation in overall size between individuals. For example, in studies of birds, this could mean including measures such as the length of the tarsus, wing, or total body mass (e.g., Bachman and Widemo 2002).

To determine which measure of body size to use in our models, we compared the fit of four generalized additive models (GAMs) for each response variable: 1) the base model with tarsus as a smoothed predictor, 2) the base model with total mass as a smoothed predictor, 3) the base model with both tarsus and mass as smoothed predictors, and 4) a model with no measure of body size or mass (i.e., the base model). In the base model the parametric (non-smooth) variables we included were sex and whether the individual was exposed to a storm in the 48 hours prior to capture (yes or no), and the smoothed variables we included were date of capture (ordinal date) and the average wet bulb temperature in the week prior to capture. The response variables were fat mass, total body water, lean mass, and hematocrit so we fit a total of 16 models (Tables S1 and S2). We chose not to include wing length as a measure of body size in our models because the grasshopper sparrows maintain a mostly terrestrial lifestyle, traversing forbs and grasses. As a result, by the breeding season (nearly a year after growing their feathers), wing feather are highly abraded and therefore their wing length is likely a poor measure of size.

We compared model fit and identified the top models using AICc. We used the packages 'mgcv' and 'AICcmodavg' to perform the analysis in R (version 4.1.1, R Core Team 2021).

Results and Conclusions

The GAMs that included mass and both mass and tarsus as predictors were considered top models for fat mass (Table S1) and the parameter estimates for each GAM predicting fat mass were consistent (Table S2). Therefore, whether a measure of body size was included or whether tarsus or mass was used did not influence our inference regarding fat mass. Similarly, there was very little difference in fit between the models predicting hematocrit (Table S1), and the estimates for the smoothed and parametric predictors were similar across models (Table S2). The top models either included no measure of body size or included mass or tarsus. Considering that hematocrit is relatively independent of body size compared to the body composition measures, it is not unexpected that controlling for body size had less impact on the model fit and output.

The top models for both lean mass and total body water included only mass, with competitive models (i.e., within 2 ΔAICc) including both mass and tarsus (Table S1). Interestingly, the relationships between weather, sex, and both total body water and lean mass depended on which body size variables were included. Specifically, GAMs that included mass and both mass and tarsus revealed that birds increased both total body water and lean mass within 48 hr of storms while GAMs that included tarsus or no measure of body size showed a

weaker relationship between storms and body composition. Thus, total body water and lean mass are sensitive to which measure of body size we include.

Overall, the GAMs that included mass were the top models predicting total body water and lean mass and were within two ΔAICc of the top model predicting fat mass and hematocrit (Table S1). Meanwhile, GAMs that included tarsus performed similarly to the base model which included no measure of body size or mass in terms of fit (Table S1) and parameter estimates (Table S2). It is possible that tarsus was not a good proxy for body size in this study population because there was very little variation between individuals in the length of the tarsus (301 of the 325 individuals were within one millimeter of the mean value of 20.1 mm). Therefore, based on model performance, we present the results from the GAMs that included mass as a predictor in the main manuscript.

Citations

Bachman G, Widemo F (1999) Relationships between body composition, body size and alternative reproductive tactics in a lekking sandpiper, the Ruff (*Philomachus pugnax*). *Functional Ecology* 13: 411-416.

Table S1. Model selection table for 16 generalized additive models (GAMs). For each response variable (fat mass, total body water, lean mass, and hematocrit), we compared the fit of four GAMs: one including just tarsus, one including total body mass, one with tarsus and total body mass, and a base model including no measure of body size.

FAT MASS

	R^2	AICc	ΔAICc
Tarsus + Mass	*0.23*	*-101.90*	-
Mass	0.23	-100.69	1.21
No measure of body size	0.10	-58.68	43.22
Tarsus	0.10	-56.62	45.28

TOTAL BODY WATER

	R^2	AICc	ΔAICc
Mass	*0.46*	*1030.09*	-
Tarsus + Mass	0.46	1031.71	1.62
Tarsus	0.34	1097.84	67.75
No measure of body size	0.33	1104.34	74.26

LEAN MASS

	R^2	AICc	ΔAICc
Mass	*0.67*	*458.35*	-
Tarsus + Mass	0.67	460.50	2.15
Tarsus	0.12	782.62	324.27
No measure of body size	0.05	801.03	342.68

HEMATOCRIT

	R^2	AICc	ΔAICc
No measure of body size	*0.07*	*-1101.08*	-
Mass	0.08	-1099.86	1.22
Tarsus	0.08	-1099.23	1.85
Tarsus + Mass	0.07	-1097.46	3.62

Table S2. Output from generalized additive models (GAMs) predicting fat mass, total body water, lean mass, and hematocrit. For each response variable, we fitted four GAMs: the base model where no measure of body size was included, the base model + tarsus, the base model + total body mass, and the base model + tarsus + total body mass. The base model included sex and whether the individual was exposed to a storm in the 48 hours prior to capture (yes or no) as parametric predictors and date of capture (ordinal date) and the average wet bulb temperature in the week prior to capture as smoothed predictors.

FAT MASS

	No measure of body size		Tarsus		Mass		Tarsus + Mass	
	Estimate	p value	Estimate	p value	Estimate	p value	Estimate	p value
**Parametric**								
Intercept	0.79 ± 0.03	<0.0001	0.79 ± 0.03	<0.0001	0.77 ± 0.03	<0.0001	0.76 ± 0.03	<0.0001
Sex	-0.15 ± 0.04	<0.0001	-0.15 ± 0.04	<0.0001	-0.12 ± 0.03	<0.001	-0.12 ± 0.03	<0.001
Storm	-0.09 ± 0.03	<0.01	-0.09 ± 0.03	<0.01	-0.08 ± 0.03	0.01	-0.08 ± 0.03	0.01
**Smooth**								
Mass	-	-	-	-	3.06	<0.0001	3.03	<0.0001
Tarsus	-	-	1.00	0.89	-	-	1.00	0.07
Temperature	1.00	0.02	1.00	0.02	1.00	0.02	1.00	0.01
Ordinal date	1.00	0.71	1.00	0.72	2.22	0.89	1.00	0.99

TOTAL BODY WATER

	No measure of body size		Tarsus		Mass		Tarsus + Mass	
	Estimate	p value	Estimate	p value	Estimate	p value	Estimate	p value
**Parametric**								
Intercept	11.92 ± 0.20	<0.0001	11.94 ± 0.20	<0.0001	11.80 ± 0.18	<0.0001	11.80 ± 0.18	<0.0001
Sex	-0.01 ± 0.22	0.98	-0.03 ± 0.22	0.91	0.11 ± 0.20	0.56	0.11 ± 0.20	0.59
Storm	0.31 ± 0.19	0.11	0.28 ± 0.19	0.13	0.44 ± 0.17	0.01	0.43 ± 0.17	0.01
**Smooth**								
Mass	-	-	-	-	1.00	<0.0001	1.00	<0.0001
Tarsus	-	-	1.00	<0.01	-	-	1.00	0.49

Temperature	1.00	0.05	1.00	0.08	1.00	0.20	1.00	0.21
Ordinal date	7.80	<0.0001	7.85	<0.0001	7.92	<0.0001	7.91	<0.0001

LEAN MASS

	No measure of body size		Tarsus		Mass		Tarsus + Mass	
	Estimate	p value	Estimate	p value	Estimate	p value	Estimate	p value
**Parametric**								
Intercept	14.12 ± 0.13	<0.0001	14.12 ± 0.12	<0.0001	14.01 ± 0.07	<0.0001	14.01 ± 0.07	<0.0001
Sex	-0.04 ± 0.13	0.76	-0.04 ± 0.13	0.75	0.06 ± 0.08	0.47	0.06 ± 0.08	0.48
Storm	-0.01 ± 0.12	0.92	-0.03 ± 0.11	0.79	0.15 ± 0.07	0.03	0.15 ± 0.07	0.03
**Smooth**								
Mass	-	-	-	-	3.67	<0.0001	3.66	<0.0001
Tarsus	-	-	2.74	<0.001	-	-	1.00	0.87
Temperature	1.00	0.20	1.00	0.27	1.00	0.79	1.00	0.80
Ordinal date	5.14	<0.01	5.23	0.01	2.22	0.05	2.21	0.05

HEMATOCRIT

	No measure of body size		Tarsus		Mass		Tarsus + Mass	
	Estimate	p value	Estimate	p value	Estimate	p value	Estimate	p value
**Parametric**								
Intercept	-0.03 ± 0.02	0.12	-0.03 ± 0.02	0.12	-0.03 ± 0.02	0.12	-0.03 ± 0.02	0.12
Sex	0.03 ± 0.02	0.16	0.03 ± 0.02	0.17	0.03 ± 0.02	0.16	0.03 ± 0.02	0.16
Storm	0.03 ± 0.02	0.05	0.03 ± 0.02	0.06	0.03 ± 0.02	0.05	0.03 ± 0.02	0.06
**Smooth**								
Mass	-	-	-	-	1.00	0.38	1.00	0.47
Tarsus	-	-	1.15	0.58	-	-	1.27	0.74
Temperature	1.77	0.58	1.88	0.52	1.82	0.56	1.89	0.52
Ordinal date	2.97	0.02	3.05	0.02	3.11	0.02	3.13	0.03
